# Adjunctive nutrients in first‐episode psychosis: A systematic review of efficacy, tolerability and neurobiological mechanisms

**DOI:** 10.1111/eip.12544

**Published:** 2018-03-21

**Authors:** Joseph Firth, Simon Rosenbaum, Philip B. Ward, Jackie Curtis, Scott B. Teasdale, Alison R. Yung, Jerome Sarris

**Affiliations:** ^1^ NICM Health Research Institute, School of Science and Health University of Western Sydney Sydney New South Wales Australia; ^2^ Division of Psychology and Mental Health University of Manchester Manchester UK; ^3^ School of Psychiatry University of New South Wales Sydney New South Wales Australia; ^4^ Black Dog Institute Prince of Wales Hospital Randwick New South Wales Australia; ^5^ Schizophrenia Research Unit Ingham Institute of Applied Medical Research Liverpool New South Wales Australia; ^6^ District Mental Health South Eastern Sydney Local Health District New South Wales Australia; ^7^ Greater Manchester Mental Health NHS Foundation Trust Manchester UK; ^8^ Department of Psychiatry University of Melbourne, The Melbourne Clinic Melbourne Victoria Australia

**Keywords:** amino acids, antioxidants, diet, early psychosis, nutrition, omega‐3

## Abstract

**Aim:**

The effects of nutrient‐based treatments, including adjunctive vitamin or antioxidant supplementation, have been explored extensively in long‐term schizophrenia. However, no systematic evaluation of trials in “first‐episode psychosis” (FEP) has been conducted, despite the potential benefits of using these treatments during the early stages of illness. Therefore, we aimed to review all studies examining efficacy, tolerability and the biological mechanisms of action, of nutrient supplementation in FEP.

**Methods:**

A systematic review of electronic databases was conducted from inception to July 2017. All information on feasibility, clinical outcomes and mechanistic findings from nutrient supplementation clinical trials was extracted and systematically synthesized.

**Results:**

Eleven studies with a total of 451 patients with FEP (from 8 independent randomized controlled trials) were eligible for inclusion. Six studies examined omega‐3 fatty acids, with inconsistent effects on psychiatric symptoms. However, mechanistic studies found significant improvements in hippocampal neuronal health and brain glutathione. Antioxidants “n‐acetyl cysteine” (*n* = 1) and vitamin C (*n* = 2) also improved oxidative status in FEP, which was associated with reduced psychiatric symptoms. No benefits were found for vitamin E (*n* = 1). Finally, one study trialling the amino acid taurine, showed significant improvements in positive symptoms and psychosocial functioning.

**Conclusion:**

There is preliminary evidence that taurine improves outcomes in FEP, whereas effects of omega‐3 and antioxidant vitamins/amino‐acids are inconsistent; perhaps mainly benefitting patients with high levels of oxidative stress. Future studies should evaluate multifaceted dietary and supplementation interventions in FEP; targeting‐specific nutritional deficits and the range of aberrant biological processes implicated in the disorder.

## 1 INTRODUCTION

First‐episode psychosis (FEP) refers to the first 2 to 5 years of a psychotic disorder, such as schizophrenia. The mainstay of treatment is antipsychotic medications, which reduce “positive symptoms” (eg, hallucinations and delusions) within weeks per month (Malla et al., 2006). However, 80% of patients relapse within 5 years (Robinson, Woerner, McMeniman, Mendelowitz, & Bilder, 2004), and only 1 in 6 achieve full recovery (Jääskeläinen et al., 2012). Furthermore, “negative symptoms” (eg, low motivation and social withdrawal) and cognitive deficits (poor memory and concentration) persist despite antipsychotic treatment, causing much of the long‐term disability associated with schizophrenia (Green, Kern, Braff, & Mintz, 2000). Therefore, to facilitate full recovery, new treatments are needed in the earliest stages of illness to reduce residual positive symptoms, and treat negative symptoms and cognitive deficits.

Nutritional deficiencies are recognized as a risk‐factor for various psychiatric disorders (Sarris, Logan, et al., 2015). People with schizophrenia generally have low‐quality diets (Dipasquale et al., 2013) and a spectrum of nutritional deficiencies, even from illness onset (Firth, Carney, et al., 2017). Furthermore, reduced levels of vitamins and polyunsaturated fatty‐acids (PUFAS) are associated with various adverse outcomes in FEP; including greater symptom severity, reduced neural integrity and neurocognitive impairments (Firth, Carney, et al., 2017; Graham et al., 2015; Shivakumar et al., 2015).

Certain food‐derived nutrients have been shown to provide effective adjunctive treatment for patients with long‐term schizophrenia. For instance, a double‐blind randomized controlled trial (RCT) in 2008 showed that 2000 mg per day of the amino‐acid “n‐acetylcysteine” (NAC) significantly reduced negative symptoms in patients with established illness (Berk et al., 2008). This finding was replicated by an independent research group in 2013 (Farokhnia et al., 2013). Additionally, our previous meta‐analysis found that adjunctive treatment with high‐dose B‐vitamins significantly reduced total psychiatric symptoms among 297 long‐term patients in 7 different studies (Firth, Stubbs, et al., 2017). The potential beneficial effects of B‐vitamins in schizophrenia has recently been confirmed by Roffman et al. (2017), who reported reductions in total and negative symptom scores from adjunctive treatment with 15 mg daily of “l‐methylfolate” (vitamin B9), along with significantly improved brain structure and connectivity after 12 weeks of treatment.

Although focused on long‐term schizophrenia, we previously found that vitamins had larger effects in patients with shorter illness duration, and those requiring lower doses of antipsychotic medications (Firth, Stubbs, et al., 2017); indicating that the benefits of vitamin supplementation may be more pronounced in early stages of psychosis. Additionally, inflammation and oxidative stress are highest in early illness (Flatow, Buckley, & Miller, 2013; Miller, Buckley, Seabolt, Mellor, & Kirkpatrick, 2011), and may drive the neurocognitive abnormalities which arise during this time (Mondelli et al., 2011). Thus, FEP may be the ideal timeframe for administering antioxidant/anti‐inflammatory nutrients as adjunctive treatments to counteract adverse neurobiological processes implicated in schizophrenia; thus reducing the likelihood of enduring symptoms and cognitive dysfunction (Chaudhry et al., 2012; Meyer, Schwarz, & Müller, 2011).

Despite the promising indications for various nutrients in FEP, the evidence for using nutrients as an adjunctive treatment for improving outcomes in this patient group has yet to be systematically evaluated. Therefore, we aimed to identify all existing RCTs of adjunctive nutrient‐based treatments in FEP, and systematically evaluate the evidence in this area. Specifically, we aimed to (1) determine the efficacy of nutrient‐based adjunctive treatments for improving patient outcomes, (2) explore the underpinning mechanisms of action by examining effects on brain volume and other biomarkers in FEP and (3) report on tolerability/adverse side‐effects of nutrient treatments.

## 2 METHODS

This systematic review and meta‐analysis followed the PRISMA statement for transparent and comprehensive reporting of methodology and results (Moher, Liberati, Tetzlaff, & Altman, 2009).

### 2.1 Search and screening process

A systematic search of electronic databases Cochrane Central Register of Controlled Trials, Health Technology Assessment Database, AMED (Allied and Complementary Medicine), HMIC, MEDLINE, PsycINFO and EMBASE was conducted from inception to July 2017. The search strategy and keyword algorithm is shown in Appendix S1, Supporting Information. All eligible studies: (1) used samples in which >75% of participants had a first episode of “psychosis,” classified according to International Classification of Diseases (ICD)/Diagnostic and Statistical Manual of Mental Disorders (DSM) criteria. Studies which did not use the term “first‐episode psychosis” were only eligible where all participants were identified as being within the first 3 years of treatment for psychotic disorders. (2) Examined effects of any nutrients including vitamins, minerals, amino‐acids, fatty‐acids (or any miscellaneous food‐based supplement), whilst herbal medicines were excluded. (3) Were controlled or open‐label human clinical trials which measured quantitative change in any physical, psychological or neurobiological outcome following nutrient‐based treatment.

### 2.2 Data extraction

The following data from each study were extracted by 2 independent authors (J.F. and S.R.):

1. *Study details*: sample size and clinical characteristics, trial design, nutrient and dosage used and length of treatment.
2. *Therapeutic target and mechanism*: the primary outcome and hypothesized operative pathway of nutrient supplementation was determined from the study aims/methods sections.
3. *Effects of adjunctive nutrients*: quantitative outcomes of nutrient supplementation on physical, psychological or neurobiological outcome measures, along with reported comparisons with any control conditions.
4. *Tolerability and adverse events*: information on trial retention, nutrient adherence and any adverse events or side‐effects which occurred during the trials.
5. *Study quality*: assessed using the Cochrane Collaboration's “Risk of Bias tool” (Higgins et al., 2011), to examine 6 aspects of trial methodology, including; randomization sequence generation, allocation concealment, blinding of participants and researchers, blinding of outcome assessments, incomplete outcome data and selective outcome reporting.

The extracted data was then synthesized for each class of nutrient examined; thus assessing the efficacy and mechanistic findings from studies examining: (1) omega‐3 PUFAs, (2) amino acids, (3) vitamins/mineral supplementation, as adjunctive treatments in FEP. Safety/tolerability data across all classes of nutrients were synthesized separately.

## 3 RESULTS

### 3.1 Overview

**Figure 1:**
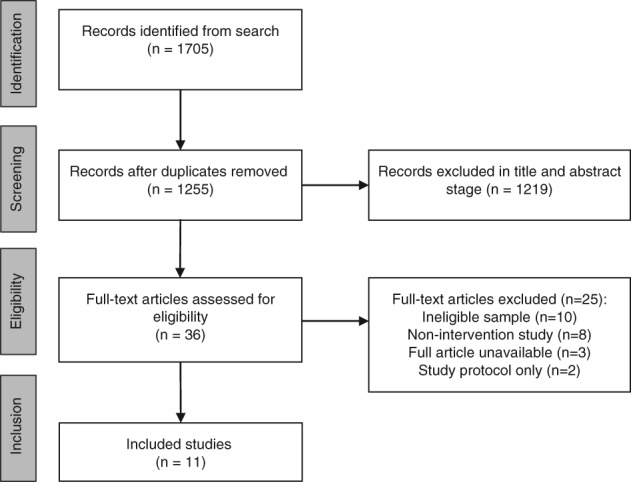
Systematic search process

Figure 1 details the study selection process. The initial database search returned 1705 results, reduced to 1255 after removing duplicates. A total of 1219 articles were excluded via title and abstract screening. Full texts were retrieved for 36 articles, of which 25 were ineligible. A further article was identified from an updated search of Google Scholar (Conus et al., 2017). Therefore, 11 studies, reporting data from 8 RCTs with 451 participants, were included in this review. All interventions examined the effects of nutrients in FEP as an adjunctive to usual treatment alone (with placebo). No studies examined different types of nutrient interventions, or compared nutrients to other forms of adjunctive therapies. Full details and findings for each study are presented in Table 1. Risk of bias summaries are presented in Appendix S2.

**Table 1:** Experimental studies of nutrient‐based adjunctive treatments for first‐episode psychosis

Study name	Exp *n* (age)	Ctrl *n* (age)	Treatment status	Nutrient details	Trial design	Therapeutic targets	Hypothesized operative pathways	Nutrient outcomes compared to control conditions	Tolerability and safety
**Omega‐3 fatty acids**									
Berger et al. (2007)	35 (21)	34 (21)	Mostly first few days of AP.	Omega‐3s 2 g EPA/day 12 wk	Double‐blind RCT	1. Symptoms (BPRS) 2. Functioning (GAF, SOFAS)	Omega‐3s modulating neurotransmission while exerting neuroprotective effects	No sustained benefits for symptoms or functioning. Omega‐3 group more likely to remit within 6 wk (15 ps vs 6 ps, *P* = .036), needed 20% lower AP doses, and experienced less EPS (*P* < .05)	Discontinued nutrient: *n* = 1 Adverse effects: none Adherence: N/R
Berger et al. (2008)	12 (20)	12 (21)	Subgroup of Berger et al. (2007) sample	Omega‐3s 2 g EPA/day 12 wk	Double‐blind RCT with flexible dose SGA	1. Brain metabolites (MRS)	Omega‐3s upregulating concentrations of neuroprotective metabolites, that is, glutamine/glutamate and GSH, which improves neural integrity and antioxidant defence	Increased bilateral glutathione (*P* = .03) and left‐hemisphere glutamate/glutamine (*P* = .049). No change in other metabolites. Increased glutathione associated with reduced negative symptoms (*r* = −.57, p = .041). Negative symptom reduction also correlated with increased trimethylamines (r = −.48, p = .025) and creatine/phosphocreatine (r = −.46, p = .032).	N/A
Wood et al. (2010)	9 (19)	8 (22)	Subgroup of Berger et al. (2007) sample	Omega‐3s 2 g EPA/day 12 wk	Double‐blind RCT with flexible dose SGA	Hippocampal neuron health (MRS)	As above; supporting neural integrity and antioxidant defence in the hippocampus	Omega‐3 treatment ameliorated the deterioration in neural health observed in placebo condition (indicated by significantly increased water in hippocampal tissues). Reduced neural health (ie, increased water in head of hippocampus) was associated with worsened negative symptoms (*r* = .49, *P* = .045).	N/A
Emsley et al. (2014)	21 (31)	12 (28)	In remission; discontinued AP after 2 to 3 years of treatment.	Omega‐3s 3 g/d + ALA 300 mg/d 24 mo	Double‐blind RCT	1. Relapse prevention 2. Symptoms (multiple) 3. Cognition (MCCB)	Omega‐3s supporting brain membrane integrity and neural processes, with ALA reducing oxidative stress and improving mitochondrial functions.	No difference in relapse rates over study period (90% exp., 75% control), relapse severity, or time to relapse (40 wk vs 38 wk, *P* = .9). No differences in psychiatric symptoms (PANSS, CGI, SOPS or CDSS) or cognition (MCCB) at monthly assessments.	Adverse effects: equal relapse in both groups Adherence: 85% in omega‐3 group
Pawelczyk, Grancow‐Grabka, Kotlicka‐Antczak, Trafalska, and Pawelczyk (2016)	36 (23)	35 (23)	Mostly <6 wk of AP. 60.6% AP naïve.	Omega‐3s 2.2 g/d 26 wk	Double‐blind RCT	1. Symptoms (PANSS)	Omega‐3 regulating many neural processes disrupted in FEP, including; neurotransmission, neuroinflammation, synaptic plasticity and neural membrane formation	Reduced total symptoms (PANSS *d* = .29, *P* = .016) 50% improvement achieved more in omega‐3 group (73.5% greater likelihood than control), *P* = .017. Reduced general symptoms (*d* = .32, *P* < .01), depression (CDSS *d* = .34, *P* < .01), CGI (*d* = .24, *P* < .05), improved GAF (*d* = .31, *P* < .05) No change in PANNS positive/negative subscales.	Discontinued nutrient: *n* = 1 Adverse effects: none Adherence: 83.2% in omega‐3 group
Pawelczyk, Grancow‐Grabka, Trafalska, Szemraj, and Pawelczyk (2017)	36 (23)	35 (23)	Subsample of Pawelczyk et al. (2016)	Omega‐3s 2.2 g/d 26 wk	Double‐blind RCT	1. Total plasma antioxidant status 2. 8‐isoprostanes F2α	Omega‐3 supplementation reversing the lipid peroxidation induced by oxidative stress in schizophrenia, in order to improve total oxidant status, and thus ameliorate symptoms.	Significantly improved total plasma antioxidant status (*d* = .22, *P* < .1) and reduced oxidative stress markers “8‐isoprostanes F2α” (*d* = .44, *P* < .01). Both associated with reduced total symptoms (CGI) and depression (CDSS) after 26‐wk of omega‐3. Reductions in 8‐isoprostanes F2α also significantly correlated with improved PANSS negative and general symptoms, along with global functioning (GAF).	Discontinued nutrient: *n* = 1 Adverse effects: none Adherence: 83.2% in omega‐3 group
**Adjunctive amino acids**									
O'Donnell et al. (2016)	61 (21)	60 (21)	Non‐acute outpatients. 66% on AP.	Taurine 4 g/d 12 wk	Double‐blind RCT	1. Symptoms (BPRS) 2. Cognition (MCCB)	Taurine modulating neurotransmission in GABA‐ and glycine‐insensitive chloride channels, inhibiting NDMA receptors, and/or activating stem cells and neural precursors to stimulate neurogenesis.	Reduced total symptoms (BPRS *d* = .67, *P* = .004), psychotic symptoms (BPRS *P* = .026, *d* = .49, PANSS positive *P* = .052, *d* = .43), depression (CDSS *d* = .44, *P* = .047), functioning (GAF *P* = .04, *d* = .46), and PANSS general (*P* = .042, *d* = .46). No change in PANSS negative, SANS or CGI. No sig change in any MCCB domain.	Discontinued nutrient; *n* = 4 Adverse effects: none Adherence: “Very good” clinician rated
Conus et al. (2017)	32	31	<5 y AP	NAC 2.7 g/d 6 mo	Double‐blind RCT	1. Symptoms (multiple) 2. Brain GSH (MRS) 3. Cognition (MCCB)	NAC upregulating brain glutathione to reduce the neuro‐inflammation and/or oxidative stress which affects parvalbumin interneurons	NAC increased brain GSH more than placebo (+23% vs −5%, *P* < .01). No significant reductions in PANSS total/subscale scores. NAC significantly reduced positive symptoms in those with high baseline oxidative stress (*P* = .02). Symptomatic improvements correlated with glutathione peroxidase activity. No difference in global cognitive improvements, but NAC significantly improved processing speed (*P* = .022)	No adverse side‐effects of NAC
**Antioxidant vitamins**									
Dakhale, Khanzode, Khanzode, and Saoji (2005)	20 (36)	20 (41)	Newly diagnosed, AP naïve outpatients.	Vitamin C 500 mg/d 8 wk	Double‐blind RCT	1. Symptoms (BPRS)	Vitamin C's antioxidant properties preventing free‐radical‐induced damage from the lipid peroxidation implicated in schizophrenia	Reduced symptoms (*P* < .01), increased plasma vitamin C (*P* < .01), reduced serum malondialdehyde (oxidative stress biomarker) (*P* < .01). Higher plasma vitamin C post‐treatment associated with greater symptomatic improvement (*r* = −.38, *P* < .05).	Discontinued nutrient: *n* = 1 Adverse effects: none Adherence: all >80%
Eranti, Gangadhar, and Janakiramaiah (1998)	12 (21)	12 (28)	AP‐naïve inpatients.	Vitamin E 3200 IU/d 2 wk	Non‐blinded RCT	1. AP side‐effects (SAS, UKU) 2. Symptoms (BPRS, CGI)	Vitamin E acting as an antioxidant to reducing haloperidol‐induced oxidative stress and thus attenuate the onset of AP side‐effects	No significant difference for AP side‐effects or psychiatric symptoms between the 2 groups.	N/R
Ingole, Belorkar, Waradkar, and Shrivastava (2011)	15 (25)	15 (25)	Newly diagnosed, AP‐naïve	Vitamin C 1000 mg/d 6 wk	Non‐blinded RCT	1. Metabolic side‐effects of olanzapine	Vitamin C's antioxidant properties attenuating the lipid/glucose dysregulation which occurs at AP treatment initiation	No significant benefits from 6 wk of vitamin C supplementation for body weight, BMI, blood sugar or lipid profile for people initiating olanzapine treatment.	N/R

Abbreviations: AP, antipsychotic medications; BPRS, brief psychiatric rating scale; CGI, clinical global impression; D, Cohen's d; GAF, global assessment of functioning; GSH, glutathione; MCCB, matrics consensus cognitive battery; MRS, magnetic resonance spectroscopy; NDMA, N‐nitrosodimethylamine; N/R, not reported; PANSS, positive and negative syndrome scale; RCT, randomized clinical trial; SANS, scale for assessment of negative symptoms; SAS, simpson angus scale; SOFAS, social and occupational functioning assessment scale; STM, short term memory; UKU, udvalg for kliniske undersøgelser.

### 3.2 Omega‐3 supplementation

Six studies with a total sample of 173 participants (from 3 double‐blind RCTs with unanimously low risk of bias) examined omega‐3 PUFAS in FEP, particularly with regards to eicosapentaenoic acid (EPA) (Berger et al., 2007; Berger et al., 2008; Emsley et al., 2014; Pawelczyk et al., 2016; Pawelczyk et al., 2017; Wood et al., 2010). All studies hypothesized that EPA supplementation would improve symptomatic outcomes due to the beneficial effects this neuroprotective PUFA has on various brain processes which are disrupted in FEP, including neuroinflammation, oxidative stress, neurotransmission and synaptic plasticity.

Initially, Berger et al. (2007) found that patients receiving first antipsychotic treatment were more likely to achieve symptomatic remission within 6 weeks when supplemented with 2 g EPA daily, rather than placebo (*P* = .036). There were no differences in remission after 12 weeks, although participants given EPA required 20% lower doses of antipsychotics. Two neuroimaging sub‐studies of these participants (*n* = 15, *n* = 24) found changes neuroprotective metabolites in the treatment groups. These studies showed omega‐3 supplementation improved hippocampal neuronal health (Wood et al., 2010) and significantly increased brain markers which reduce oxidative stress and neural damage, such as glutathione (Berger et al., 2008). Furthermore, both improved hippocampal neuronal health and increased glutathione were correlated with reduced negative symptoms (*P* = .041 and .045, respectively).

Pawelczyk et al. (2016) trialled 2.2 mg omega‐3 per day (containing 1320 mg EPA plus 880 mg docosahexaenoic acid [DHA]) as an adjunctive to initial antipsychotic treatment, over a period of 26 weeks in 71 patients with FEP. This showed that adjunctive treatment with EPA/DHA omega‐3 improved total symptoms, functioning and depression significantly more than placebo (olive oil). A sub‐study of the same sample investigated how changes in blood markers of oxidative stress moderated omega‐3 treatment outcomes (Pawelczyk et al., 2017). This found omega‐3 increased total plasma antioxidant capacity (*P* < .001) and reduced oxidative marker “8‐isoprostane F2α” (*P* < .001). Correlation analyses further showed that improvements in total plasma antioxidant capacity from omega‐3 treatment were significantly associated with reductions in total psychiatric symptoms (clinical global impression‐severity [CGI‐S]) and measures of depression. Additionally, reductions in blood levels of 8‐isoprostane F2α held significant correlations with reductions in depression, negative symptoms and functional disability following the intervention.

Finally, Emsley et al. (2014) used a combination of omega‐3 (3 g daily) with alpha‐linoleic acid (ALA) to prevent relapse during antipsychotic discontinuation in remitted FEP. This was ineffective; with equivalent relapse rates in both omega‐3 and placebo conditions over 2 years.

### 3.3 Adjunctive amino acids

Two studies have examined amino acids, taurine and NAC as adjunctive treatments in FEP: O'Donnell et al. (2016) hypothesized that taurine's actions as a peptide neurotransmitter, N‐nitrosodimethylamine (NDMA)‐receptor antagonist and neuroprotective agent would reduce both psychiatric symptoms and cognitive impairments. The high‐quality RCT found that after 12 weeks, 4 g/d of adjunctive taurine (*n* = 47) significantly improved total symptoms, psychotic symptoms, depression and functioning more than placebo (*n* = 39), however, there were no significant effects on cognition.

Conus et al. (2017) reported results from a double‐blind RCT of NAC in 63 patients, hypothesizing that this antioxidant would reduce the neuroinflammation and oxidative stress that may impair functioning of parvalbumin interneurons in FEP. Although 2700 mg/d of NAC supplementation significantly increased brain levels of the antioxidant glutathione after 6 months, there was no overall beneficial effect on symptoms compared to placebo. However, significantly greater reductions in psychotic symptoms were observed among those with higher levels of baseline oxidative stress (*P* = .02). Additionally, despite no effect on global cognition, NAC significantly improved cognitive processing speed.

### 3.4 Antioxidant vitamins

Three studies have used adjunctive vitamins in newly diagnosed, antipsychotic‐naïve FEP patients. All hypothesized beneficial effects would occur from the antioxidant properties of the vitamins used. Dakhale et al. (2005) examined Vitamin C's (500 mg/d for 8 weeks) effects on lipid peroxidation and symptoms in FEP. Vitamin C significantly reduced total symptoms, and reductions were correlated with higher levels of plasma Vitamin C post‐treatment (*r* = −.38, *P* < .05). Vitamin C also reduced malondialdehyde (a serum marker of lipid peroxidation) significantly more than placebo conditions (*P* < .01), although this was not correlated with symptomatic improvements.

Ingole et al. (2011) examined if the antioxidant properties of vitamin C could also ameliorate the metabolic side‐effects of antipsychotic treatment. In an open‐label trial among patients undergoing their first antipsychotic treatment, they compared metabolic outcomes of olanzapine plus 1000 mg/d of vitamin C to olanzapine alone. However, the study found no effect of vitamin C on metabolic outcomes, with equivalent weight‐gain and lipid dysregulation occurring from olanzapine treatment in both groups. Eranti et al. (1998) administered vitamin E (3200 IU/d) in an open‐label study, in order to counteract haloperidol‐induced oxidative stress and thus reduce extrapyramidal symptoms. However, vitamin E had no significant effect on either symptoms or antipsychotic side‐effects.

### 3.5 Side‐effects and adverse events

Across all 3 RCTs of PUFA in FEP (Berger et al., 2007; Emsley et al., 2014; Pawelczyk et al., 2016), there were no reports of adverse events attributable to omega‐3 treatment. Furthermore, feasibility data indicated high tolerability, as retention was consistently high and adherence was >80%. Both of the trials of taurine (O'Donnell et al., 2016) and NAC (Conus et al., 2017) also reported that there were no adverse side‐effects from either of these amino acids in FEP. Similarly, adjunctive treatment with antioxidant vitamins (C and E) did not result in any adverse events or side‐effects (Dakhale et al., 2005; Eranti et al., 1998). Thus, across all studies to date, there is no indication of adjunctive nutrients causing negative side‐effects or increasing adverse events in FEP.

## 4 DISCUSSION

This review aimed to assess the efficacy, tolerability and biological mechanisms of action for nutrient‐based compounds trialled as adjunctive treatments for FEP to date. Across the 11 studies (with a total of 451 participants) that were identified, there are some encouraging findings for certain nutrient‐based adjunctive treatments in FEP.

Omega‐3 is the most widely studied nutrient in FEP to date. The benefits of omega‐3 supplementation has been studied across a broad range of neurodegenerative and psychiatric disorders (Fotuhi, Mohassel, & Yaffe, 2009; Sarris et al., 2016). However, the presumed link between PUFA consumption and brain health has yet to be fully confirmed (Bos, van Montfort, Oranje, Durston, & Smeets, 2016). Our review of 6 studies (from 3 independent RCTs) found that the evidence for using omega‐3 in the treatment of FEP is equivocal; as is the case in both long‐term schizophrenia, and in those at ultra‐high risk of developing psychosis (Amminger, Harris, McGorry, & Henry, 2013; Amminger, Schäfer, Schlögelhofer, Klier, & McGorry, 2015; Fusar‐Poli & Berger, 2012; McGorry et al., 2017). Although 2 trials found no benefits for people with FEP (Berger et al., 2007; Emsley et al., 2014), one study using an extended course of supplementation (24 weeks, compared to 12 weeks in Berger et al., 2007) observed significant improvements in both symptoms and real‐world functioning (Pawelczyk et al., 2016). Additionally, the 3 mechanistic sub‐studies from these trials consistently found significant reductions in central markers of neuronal oxidative damage (Berger et al., 2008; Wood et al., 2010) and peripheral markers of lipid peroxidation/oxidative stress (Pawelczyk et al., 2017). Furthermore, there appears to be a relationship between these brain and biomarker improvements with reduced negative symptoms (Berger et al., 2008; Pawelczyk et al., 2017; Wood et al., 2010). Thus, more research is required to assess the longer‐term benefits of omega‐3 supplementation in FEP, and to determine if certain patient subgroups (ie, those most affected by negative symptoms and oxidative stress and inflammation) may be more responsive to such treatments (Rapaport et al., 2016).

Two further trials found that the dietary antioxidants NAC and vitamin C were also effective for improving markers of central and peripheral stress in FEP (Conus et al., 2017; Dakhale et al., 2005). However, larger studies are required to examine how these reductions in oxidative stress may relate to clinical improvements. This is a promising avenue for future research, given that RCTs of other anti‐inflammatory (non‐nutrient) agents have also demonstrated efficacy as adjunctive treatments in schizophrenia and FEP (Solmi et al., 2017; Sommer et al., 2013), specifically for reducing negative symptoms and cognitive deficits, which is a major unmet need in FEP (Meyer et al., 2011).

However, the positive findings of the high‐quality and modestly sized taurine trial (O'Donnell et al., 2016) suggests the benefits of nutrient supplementation in FEP may potentially extend beyond antioxidant/anti‐inflammatory effects. Taurine is posited to act through other mechanisms to improve brain health in FEP, such as via NDMA receptor inhibition and stem cell activation (O'Donnell et al., 2016). In consideration of this, symptomatic improvements from taurine supplementation were observed for positive, rather than negative symptoms, which are often linked to inflammation/oxidative stress. Along with attempting to replicate the effects observed from taurine, future research should also investigate the potential benefits of other neuroprotective nutrients in FEP. Cross‐sectional research indicates that folate and vitamin D are worthy candidates for adjunctive treatment trials, given that people with FEP have particularly low circulating levels for both of these in comparison to age‐ and gender‐matched controls (Firth, Carney, et al., 2017), even when controlling for other factors such as dietary nutrition intake (Kale et al., 2010). Furthermore, lower levels of both folate and vitamin D among patients with FEP have been linked to more severe negative symptoms (Song et al., 2014; Yee, See, Abdul Rashid, Neelamekam, & Lee, 2016), indicating that resolving these deficiencies may improve clinical outcomes. Indeed, folate supplementation in long‐term schizophrenia has recently been found to reduce negative symptoms while improving brain structure and connectivity (Roffman et al., 2017). Although experimental studies of vitamin D are currently lacking, the plausible role that prenatal and perinatal deficiency has in the aetiology of psychotic disorders (McGrath et al., 2004; McGrath, Eyles, Mowry, Yolken, & Buka, 2003), along with known serotonergic actions of vitamin D, present a strong case for supplementation trials in schizophrenia (Patrick & Ames, 2015). An ongoing RCT will soon shed new insights into the benefits of this potentially neurosteroidal compound in the treatment of FEP (ISRCTN: 12424842).

One limitation of our findings is that, despite the numerous small‐scale studies, there are currently no large‐scale, multisite RCTs of any individual nutrients in FEP to date. Furthermore, there was insufficient homogeneity across study interventions/outcomes to pool existing data using meta‐analytic techniques. Thus, the encouraging findings from single positive studies must be shown to be replicable before these nutrients can be recommended for clinical use as adjunctive treatments in FEP. However, a further limitation of the literature is that all studies to date have focused on single‐nutrient treatments. Future studies may benefit from moving beyond single‐nutrient trials, since research in other populations suggests that combining beneficial nutrients within multi‐nutrient formulas may result in greater benefits than single‐nutrient supplementation, and is less likely to cause nutritional imbalances (Kaplan, Rucklidge, Romijn, & Dolph, 2015; Oulhaj, Jernerén, Refsum, Smith, & de Jager, 2016; Rucklidge, Johnstone, & Kaplan, 2013). There is increasing interest in using multi‐nutrients to target mechanistic pathways implicated in neuropsychiatric conditions (Dean et al., 2015; Oulhaj et al., 2016; Sarris, Stough, et al., 2015; Sarris, Logan, et al., 2015). Although this approach has yet to be trialled in schizophrenia, a combined nutrient formula which simultaneously reduces damaging oxidative processes, while restoring deficiencies (if apparent) in folate and vitamin D could provide effective adjunctive treatment for psychotic disorders; particularly during FEP, as oxidative stress is greatest at this point (Flatow et al., 2013). Furthermore, younger patients may be more responsive to nutrient treatments (Firth, Stubbs, et al., 2017).

Across all of our included studies, there was no indication of any harmful side‐effects, harmful interactions with antipsychotic medications or increased adverse events from the nutrients trialled in FEP. This suggests that continued research into the benefits of nutrient‐based adjunctive treatments for FEP is worthwhile and ethical.

However, beyond nutrient supplementation, it is important to also consider the dietary nutrition intake of people with FEP. To date, the bulk of the research on dietary factors in schizophrenia has focused on reducing over‐consumption of obesogenic foods, and preventing the associated weight gain (Teasdale, Ward, Rosenbaum, Samaras, & Stubbs, 2016). Future studies are required to examine how inadequate nutrient intake influences outcomes of FEP, as this could feasibly contribute towards poor recovery rates observed in this population (Teasdale, Ward, Rosenbaum, Watkins, et al., 2016). For instance, depriving the brain of neuroprotective nutrients may exacerbate psychiatric symptoms (Goff et al., 2004) whereas consuming nutritionally devoid high‐fat foods increases systematic inflammation and oxidative stress (Devaraj, Wang‐Polagruto, Polagruto, Keen, & Jialal, 2008; Lundman et al., 2007). Further, an RCT in patients with major depression has recently shown significant reductions in psychiatric symptoms from corrective dietary intervention (Jacka et al., 2017). Clearly, additional supplementation trials and dietary interventions are warranted to establish how nutrition‐based treatments can attenuate the cardiometabolic dysfunction and poor psychosocial recovery associated with antipsychotic treatment; in order to improve physical, psychiatric and neurobiological outcomes from earliest stages of this illness.

## Supporting information


**Appendix S1.** Systematic search algorithm.
**Appendix S2.** Cochrane risk of bias summaries for included studies.

## References

[eip12544-bib-0001] Amminger, G. P. , Harris, M. S. , McGorry, P. D. , & Henry, L. P. (2013). Omega‐3 fatty acids for indicated prevention: Treatment results and pathomechanisms. European Archives of Psychiatry and Clinical Neuroscience, 1, S44–S45.

[eip12544-bib-0002] Amminger, G. P. , Schäfer, M. R. , Schlögelhofer, M. , Klier, C. M. , & McGorry, P. D. (2015). Longer‐term outcome in the prevention of psychotic disorders by the Vienna omega‐3 study. Nature Communications, 6, 7934. 10.1038/ncomms8934 PMC4918317 26263244

[eip12544-bib-0003] Berger, G. E. , Proffitt, T. M. , McConchie, M. , Yuen, H. , Wood, S. J. , Amminger, G. P. , … McGorry, P. D. (2007). Ethyl‐eicosapentaenoic acid in first‐episode psychosis: A randomized, placebo‐controlled trial. Journal of Clinical Psychiatry, 68, 1867–1875. 18162017 10.4088/jcp.v68n1206

[eip12544-bib-0004] Berger, G. E. , Wood, S. J. , Wellard, R. M. , Proffitt, T. M. , McConchie, M. , Amminger, G. P. , … McGorry, P. D. (2008). Ethyl‐eicosapentaenoic acid in first‐episode psychosis. A 1H‐MRS study. Neuropsychopharmacology, 33, 2467–2473. 18199999 10.1038/sj.npp.1301628

[eip12544-bib-0005] Berk, M. , Copolov, D. , Dean, O. , Lu, K. , Jeavons, S. , Schapkaitz, I. , … Bush, A. I. (2008). N‐acetyl cysteine as a glutathione precursor for schizophrenia – A double‐blind, randomized, placebo‐controlled trial. Biological Psychiatry, 64, 361–368. 18436195 10.1016/j.biopsych.2008.03.004

[eip12544-bib-0006] Bos, D. J. , van Montfort, S. J. , Oranje, B. , Durston, S. , & Smeets, P. A. (2016). Effects of omega‐3 polyunsaturated fatty acids on human brain morphology and function: What is the evidence? European Neuropsychopharmacology, 26, 546–561. 26742901 10.1016/j.euroneuro.2015.12.031

[eip12544-bib-0007] Chaudhry, I. B. , Hallak, J. , Husain, N. , Minhas, F. , Stirling, J. , Richardson, P. , … Deakin, B. (2012). Minocycline benefits negative symptoms in early schizophrenia: A randomised double‐blind placebo‐controlled clinical trial in patients on standard treatment. Journal of Psychopharmacology, 26, 1185–1193. 22526685 10.1177/0269881112444941

[eip12544-bib-0008] Conus, P. , Seidman, L. J. , Fournier, M. , Xin, L. , Cleusix, M. , Baumann, P. S. , … Do, K. Q. (2017). N‐acetylcysteine in a double‐blind randomized placebo‐controlled trial: Toward biomarker‐guided treatment in early psychosis. Schizophrenia Bulletin, *44*, 317–327. 10.1093/schbul/sbx093 PMC5815074 29462456

[eip12544-bib-0009] Dakhale, G. N. , Khanzode, S. D. , Khanzode, S. S. , & Saoji, A. (2005). Supplementation of vitamin C with atypical antipsychotics reduces oxidative stress and improves the outcome of schizophrenia. Psychopharmacology, 182, 494–498. 16133138 10.1007/s00213-005-0117-1

[eip12544-bib-0010] Dean, O. M. , Turner, A. , Malhi, G. S. , Ng, C. , Cotton, S. M. , Dodd, S. , … Berk, M. (2015). Design and rationale of a 16‐week adjunctive randomized placebo‐controlled trial of mitochondrial agents for the treatment of bipolar depression. Revista Brasileira de Psiquiatria, 37, 03–12. 10.1590/1516-4446-2013-1341 25295681

[eip12544-bib-0011] Devaraj, S. , Wang‐Polagruto, J. , Polagruto, J. , Keen, C. L. , & Jialal, I. (2008). High‐fat, energy‐dense, fast‐food–style breakfast results in an increase in oxidative stress in metabolic syndrome. Metabolism, 57, 867–870. 18502272 10.1016/j.metabol.2008.02.016 PMC2692901

[eip12544-bib-0012] Dipasquale, S. , Pariante, C. M. , Dazzan, P. , Aguglia, E. , McGuire, P. , & Mondelli, V. (2013). The dietary pattern of patients with schizophrenia: A systematic review. Journal of Psychiatric Research, 47, 197–207. 23153955 10.1016/j.jpsychires.2012.10.005

[eip12544-bib-0013] Emsley, R. , Chiliza, B. , Asmal, L. , du Plessis, S. , Phahladira, L. , van Niekerk, E. , … Harvey, B. H. (2014). A randomized, controlled trial of omega‐3 fatty acids plus an antioxidant for relapse prevention after antipsychotic discontinuation in first‐episode schizophrenia. Schizophrenia Research, 158, 230–235. 24996507 10.1016/j.schres.2014.06.004

[eip12544-bib-0014] Eranti, V. S. , Gangadhar, B. N. , & Janakiramaiah, N. (1998). Haloperidol‐induced extrapyramidal reaction: Lack of protective effect by vitamin E. Psychopharmacology, 140, 418–420. 9888616 10.1007/s002130050784

[eip12544-bib-0015] Farokhnia, M. , Azarkolah, A. , Adinehfar, F. , Khodaie‐Ardakani, M.‐R. , Yekehtaz, H. , Tabrizi, M. , Akhondzadeh, S. (2013). N‐acetylcysteine as an adjunct to risperidone for treatment of negative symptoms in patients with chronic schizophrenia: A randomized, double‐blind, placebo‐controlled study. Clinical Neuropharmacology, 36, 185–192. 24201233 10.1097/WNF.0000000000000001

[eip12544-bib-0016] Firth, J. , Carney, R. , Stubbs, B. , Teasdale, S. , Vancampfort, D. , Ward, P. , … Sarris, J. (2017). Nutritional deficiencies and clinical correlates in first‐episode psychosis: A systematic review and meta‐analysis. Schizophrenia Bulletin. 10.1093/schbul/sbx162 PMC6192507 29206972

[eip12544-bib-0017] Firth, J. , Stubbs, B. , Sarris, J. , Rosenbaum, S. , Teasdale, S. , Berk, M. , & Yung, A. (2017). The effects of vitamin and mineral supplementation on symptoms of schizophrenia: A systematic review and meta‐analysis. Psychological Medicine, *47*, 1515–1527. 10.1017/S0033291717000022 28202095

[eip12544-bib-0018] Flatow, J. , Buckley, P. , & Miller, B. J. (2013). Meta‐analysis of oxidative stress in schizophrenia. Biological Psychiatry, 74, 400–409. 23683390 10.1016/j.biopsych.2013.03.018 PMC4018767

[eip12544-bib-0019] Fotuhi, M. , Mohassel, P. , & Yaffe, K. (2009). Fish consumption, long‐chain omega‐3 fatty acids and risk of cognitive decline or Alzheimer disease: A complex association. Nature Clinical Practice Neurology, 5, 140–152. 10.1038/ncpneuro1044 19262590

[eip12544-bib-0020] Fusar‐Poli, P. , & Berger, G. (2012). Eicosapentaenoic acid interventions in schizophrenia: Meta‐analysis of randomized, placebo‐controlled studies. Journal of Clinical Psychopharmacology, 32, 179–185. 22367656 10.1097/JCP.0b013e318248b7bb

[eip12544-bib-0021] Goff, D. C. , Bottiglieri, T. , Arning, E. , Shih, V. , Freudenreich, O. , Evins, A. E. , … Coyle, J. (2004). Folate, homocysteine, and negative symptoms in schizophrenia. American Journal of Psychiatry, 161, 1705–1708. 15337665 10.1176/appi.ajp.161.9.1705

[eip12544-bib-0022] Graham, K. A. , Keefe, R. S. , Lieberman, J. A. , Calikoglu, A. S. , Lansing, K. M. , & Perkins, D. O. (2015). Relationship of low vitamin D status with positive, negative and cognitive symptom domains in people with first‐episode schizophrenia. Early Intervention in Psychiatry, 9, 397–405. 24612563 10.1111/eip.12122

[eip12544-bib-0023] Green, M. F. , Kern, R. S. , Braff, D. L. , & Mintz, J. (2000). Neurocognitive deficits and functional outcome in schizophrenia: Are we measuring the" right stuff"? Schizophrenia Bulletin, 26, 119–136. 10755673 10.1093/oxfordjournals.schbul.a033430

[eip12544-bib-0024] Higgins, J. P. , Altman, D. G. , Gøtzsche, P. C. , Jüni, P. , Moher, D. , Oxman, A. D. , et al. (2011). The Cochrane Collaboration's tool for assessing risk of bias in randomised trials. BMJ, 343, d5928. 22008217 10.1136/bmj.d5928 PMC3196245

[eip12544-bib-0025] Ingole, S. , Belorkar, N. , Waradkar, P. , & Shrivastava, M. (2011). Role of ascorbic acid supplementation on prevention of olanzapine induced metabolic side effects in schizophrenic patients. Indian Journal of Public Health Research & Development, 2, 12–16.

[eip12544-bib-0026] Jääskeläinen, E. , Juola, P. , Hirvonen, N. , McGrath, J. J. , Saha, S. , Isohanni, M. , … Miettunen, J. (2012). A systematic review and meta‐analysis of recovery in schizophrenia. Schizophrenia Bulletin, 39, 1296–1306. 23172003 10.1093/schbul/sbs130 PMC3796077

[eip12544-bib-0027] Jacka, F. N. , O'Neil, A. , Opie, R. , Itsiopoulos, C. , Cotton, S. , Mohebbi, M. , … Berk, M. (2017). A randomised controlled trial of dietary improvement for adults with major depression (the ‘SMILES'trial). BMC Medicine, 15, 23. 28137247 10.1186/s12916-017-0791-y PMC5282719

[eip12544-bib-0028] Kale, A. , Naphade, N. , Sapkale, S. , Kamaraju, M. , Pillai, A. , Joshi, S. , & Mahadik, S. (2010). Reduced folic acid, vitamin B<inf>12</inf> and docosahexaenoic acid and increased homocysteine and cortisol in never‐medicated schizophrenia patients: Implications for altered one‐carbon metabolism. Psychiatry Research, 175, 47–53. 19969375 10.1016/j.psychres.2009.01.013

[eip12544-bib-0029] Kaplan, B. J. , Rucklidge, J. J. , Romijn, A. R. , & Dolph, M. (2015). A randomised trial of nutrient supplements to minimise psychological stress after a natural disaster. Psychiatry Research, 228, 373–379. 26154816 10.1016/j.psychres.2015.05.080

[eip12544-bib-0030] Lundman, P. , Boquist, S. , Samnegård, A. , Bennermo, M. , Held, C. , Ericsson, C.‐G. , … Tornvall, P. (2007). A high‐fat meal is accompanied by increased plasma interleukin‐6 concentrations. Nutrition, Metabolism, and Cardiovascular Diseases, 17, 195–202. 10.1016/j.numecd.2005.11.009 17367705

[eip12544-bib-0031] Malla, A. , Norman, R. , Schmitz, N. , Manchanda, R. , BÉChard‐Evans, L. , Takhar, J. , & Haricharan, R. (2006). Predictors of rate and time to remission in first‐episode psychosis: A two‐year outcome study. Psychological Medicine, 36, 649–658. 16515734 10.1017/S0033291706007379

[eip12544-bib-0032] McGorry, P. D. , Nelson, B. , Markulev, C. , Yuen, H. P. , Schafer, M. R. , Mossaheb, N. , et al. (2017). Effect of omega‐3 polyunsaturated fatty acids in young people at ultrahigh risk for psychotic disorders: The NEURAPRO randomized clinical trial. JAMA Psychiatry, 74, 19–27. 27893018 10.1001/jamapsychiatry.2016.2902

[eip12544-bib-0033] McGrath, J. , Eyles, D. , Mowry, B. , Yolken, R. , & Buka, S. (2003). Low maternal vitamin D as a risk factor for schizophrenia: A pilot study using banked sera. Schizophrenia Research, 63, 73–78. 12892860 10.1016/s0920-9964(02)00435-8

[eip12544-bib-0034] McGrath, J. , Saari, K. , Hakko, H. , Jokelainen, J. , Jones, P. , Järvelin, M.‐R. , … Isohanni, M. (2004). Vitamin D supplementation during the first year of life and risk of schizophrenia: A Finnish birth cohort study. Schizophrenia Research, 67, 237–245. 14984883 10.1016/j.schres.2003.08.005

[eip12544-bib-0035] Meyer, U. , Schwarz, M. J. , & Müller, N. (2011). Inflammatory processes in schizophrenia: A promising neuroimmunological target for the treatment of negative/cognitive symptoms and beyond. Pharmacology & Therapeutics, 132, 96–110. 21704074 10.1016/j.pharmthera.2011.06.003

[eip12544-bib-0036] Miller, B. J. , Buckley, P. , Seabolt, W. , Mellor, A. , & Kirkpatrick, B. (2011). Meta‐analysis of cytokine alterations in schizophrenia: Clinical status and antipsychotic effects. Biological Psychiatry, 70, 663–671. 21641581 10.1016/j.biopsych.2011.04.013 PMC4071300

[eip12544-bib-0037] Moher, D. , Liberati, A. , Tetzlaff, J. , & Altman, D. G. (2009). Preferred reporting items for systematic reviews and meta‐analyses: The PRISMA statement. Annals of Internal Medicine, 151, 264–269. 19622511 10.7326/0003-4819-151-4-200908180-00135

[eip12544-bib-0038] Mondelli, V. , Cattaneo, A. , Murri, M. B. , Di Forti, M. , Handley, R. , Hepgul, N. , et al. (2011). Stress and inflammation reduce BDNF expression in first‐episode psychosis: A pathway to smaller hippocampal volume. The Journal of Clinical Psychiatry, 72, 1677–1684. 21672499 10.4088/JCP.10m06745 PMC4082665

[eip12544-bib-0039] O'Donnell, C. P. , Allott, K. A. , Murphy, B. P. , Yuen, H. P. , Proffitt, T.‐M. , Papas, A. , et al. (2016). Adjunctive Taurine in first‐episode psychosis: A phase 2, double‐blind, randomized, placebo‐controlled study. The Journal of Clinical Psychiatry, 77, e1610–e1617. 27835719 10.4088/JCP.15m10185

[eip12544-bib-0040] Oulhaj, A. , Jernerén, F. , Refsum, H. , Smith, A. D. , & de Jager, C. A. (2016). Omega‐3 fatty acid status enhances the prevention of cognitive decline by B vitamins in mild cognitive impairment. Journal of Alzheimer's Disease, 50, 547–557. 10.3233/JAD-150777 PMC4927899 26757190

[eip12544-bib-0041] Patrick, R. P. , & Ames, B. N. (2015). Vitamin D and the omega‐3 fatty acids control serotonin synthesis and action, part 2: Relevance for ADHD, bipolar disorder, schizophrenia, and impulsive behavior. The FASEB Journal, 29, 2207–2222. 25713056 10.1096/fj.14-268342

[eip12544-bib-0042] Pawelczyk, T. , Grancow‐Grabka, M. , Kotlicka‐Antczak, M. , Trafalska, E. , & Pawelczyk, A. (2016). A randomized controlled study of the efficacy of six‐month supplementation with concentrated fish oil rich in omega‐3 polyunsaturated fatty acids in first episode schizophrenia. Journal of Psychiatric Research, 73, 34–44. 26679763 10.1016/j.jpsychires.2015.11.013

[eip12544-bib-0043] Pawelczyk, T. , Grancow‐Grabka, M. , Trafalska, E. , Szemraj, J. , & Pawelczyk, A. (2017). Oxidative stress reduction related to the efficacy of n‐3 polyunsaturated fatty acids in first episode schizophrenia: Secondary outcome analysis of the OFFER randomized trial. Prostaglandins, Leukotrienes, and Essential Fatty Acids, 121, 7–13. 10.1016/j.plefa.2017.05.004 28651701

[eip12544-bib-0044] Rapaport, M. H. , Nierenberg, A. A. , Schettler, P. J. , Kinkead, B. , Cardoos, A. , Walker, R. , & Mischoulon, D. (2016). Inflammation as a predictive biomarker for response to omega‐3 fatty acids in major depressive disorder: A proof‐of‐concept study. Molecular Psychiatry, 21, 71–79. 25802980 10.1038/mp.2015.22 PMC4581883

[eip12544-bib-0045] Robinson, D. G. , Woerner, M. G. , McMeniman, M. , Mendelowitz, A. , & Bilder, R. M. (2004). Symptomatic and functional recovery from a first episode of schizophrenia or schizoaffective disorder. American Journal of Psychiatry, 161(3), 473–479. 14992973 10.1176/appi.ajp.161.3.473

[eip12544-bib-0046] Roffman, J. , Petruzzi, L. , Tanner, A. , Brown, H. , Eryilmaz, H. , Ho, N. , et al. (2017). Biochemical, physiological and clinical effects of l‐methylfolate in schizophrenia: A randomized controlled trial. Molecular Psychiatry, *23*, 316–322. 10.1038/mp.2017.41 PMC5599314 28289280

[eip12544-bib-0047] Rucklidge, J. J. , Johnstone, J. , & Kaplan, B. J. (2013). Magic bullet thinking‐why do we continue to perpetuate this fallacy? The British Journal of Psychiatry, 203, 154–154. 10.1192/bjp.203.2.154 23908343

[eip12544-bib-0048] Sarris, J. , Logan, A. C. , Akbaraly, T. N. , Amminger, G. P. , Balanzá‐Martínez, V. , Freeman, M. P. , … International Society for Nutritional Psychiatry Research . (2015). Nutritional medicine as mainstream in psychiatry. The Lancet. Psychiatry, 2, 271–274. 26359904 10.1016/S2215-0366(14)00051-0

[eip12544-bib-0049] Sarris, J. , Murphy, J. , Mischoulon, D. , Papakostas, G. I. , Fava, M. , Berk, M. , & Ng, C. H. (2016). Adjunctive nutraceuticals for depression: A systematic review and meta‐analyses. American Journal of Psychiatry, 173, 575–587. 27113121 10.1176/appi.ajp.2016.15091228

[eip12544-bib-0050] Sarris, J. , Stough, C. , Bousman, C. , Murphy, J. , Savage, K. , Smith, D. J. , … Mischoulon, D. (2015). An adjunctive antidepressant nutraceutical combination in treating major depression: Study protocol, and clinical considerations. Advances in Integrative Medicine, 2, 49–55.

[eip12544-bib-0051] Shivakumar, V. , Kalmady, S. V. , Amaresha, A. C. , Jose, D. , Narayanaswamy, J. C. , Agarwal, S. M. , … Gangadhar, B. N. (2015). Serum vitamin D and hippocampal gray matter volume in schizophrenia. Psychiatry Research: Neuroimaging, 233, 175–179. 10.1016/j.pscychresns.2015.06.006 26163386

[eip12544-bib-0052] Solmi, M. , Veronese, N. , Thapa, N. , Facchini, S. , Stubbs, B. , Fornaro, M. , … Correll, C. U. (2017). Systematic review and meta‐analysis of the efficacy and safety of minocycline in schizophrenia. CNS Spectrums, *22*, 415–426. 10.1017/S1092852916000638 28181901

[eip12544-bib-0053] Sommer, I. E. , van Westrhenen, R. , Begemann, M. J. , de Witte, L. D. , Leucht, S. , & Kahn, R. S. (2013). Efficacy of anti‐inflammatory agents to improve symptoms in patients with schizophrenia: An update. Schizophrenia Bulletin, 40(1), 181–191. 24106335 10.1093/schbul/sbt139 PMC3885306

[eip12544-bib-0054] Song, X. , Fan, X. , Li, X. , Kennedy, D. , Pang, L. , Quan, M. , … Lv, L. (2014). Serum levels of BDNF, folate and homocysteine: In relation to hippocampal volume and psychopathology in drug naive, first episode schizophrenia. Schizophrenia Research, 159, 51–55. 25128453 10.1016/j.schres.2014.07.033

[eip12544-bib-0055] Teasdale, S. B. , Ward, P. B. , Rosenbaum, S. , Samaras, K. , & Stubbs, B. (2016). Solving a weighty problem: Systematic review and meta‐analysis of nutrition interventions in severe mental illness. The British Journal of Psychiatry, 210(2), 110–118. 27810893 10.1192/bjp.bp.115.177139

[eip12544-bib-0056] Teasdale, S. B. , Ward, P. B. , Rosenbaum, S. , Watkins, A. , Curtis, J. , Kalucy, M. , & Samaras, K. (2016). A nutrition intervention is effective in improving dietary components linked to cardiometabolic risk in youth with first‐episode psychosis. British Journal of Nutrition, 115, 1987–1993. 27153205 10.1017/S0007114516001033

[eip12544-bib-0057] Wood, S. J. , Cocchi, L. , Proffitt, T. M. , McConchie, M. , Jackson, G. D. , Takahashi, T. , … Berger, G. E. (2010). Neuroprotective effects of ethyl‐eicosapentaenoic acid in first episode psychosis: A longitudinal T2 relaxometry pilot study. Psychiatry Research: Neuroimaging, 182, 180–182. 10.1016/j.pscychresns.2009.12.003 20413278

[eip12544-bib-0058] Yee, J. Y. , See, Y. M. , Abdul Rashid, N. A. , Neelamekam, S. , & Lee, J. (2016). Association between serum levels of bioavailable vitamin D and negative symptoms in first‐episode psychosis. Psychiatry Research, 243, 390–394. 27449008 10.1016/j.psychres.2016.07.003

