# Supplementary material for: Adjunctive nutrients in first‐episode psychosis: A systematic review of efficacy, tolerability and neurobiological mechanisms
Source: Early Interv Psychiatry. 2018 Mar 21;12(5):774–83. doi: 10.1111/eip.12544 (PMC6175456; doi:10.1111/eip.12544)

**Appendices**

Supplement 1. Systematic search algorithm

| ***Category*** | **Search words used (‘OR’ terms)** |
| --- | --- |
| *Population* | Schizoaffective; schizophr*; Psychosis; Psychotic; Psychoses; Severe mental; Serious mental; Antipsychotic*  AND  Naïve; first episode; first onset; early onset; early intervention; young; youth; Early psychosis; adolescent |
| *Intervention* | Vitamin*; Mineral*; Tocopherol; Alphatocopherol; Carotene; Retinol; Thiamine; Riboflavin; Niacin; Niacinamide; Nicotinic Acid; Pantothenic; Pyridox*; Biotin; Methylfolate; Folate; Folinic Acid; Folic acid; Inositol; Cyanocobalamin; Methylcobalamin; Cobalamin; Ascorbic Acid; Cholecalciferol; Tocopherols; Trace Element; Calcium; Phosphorus; Magnesium; Potassium; Manganese; Zinc; Selenium; Boron; Chromium; Lycopene; Isoflav*; Flavonoid*; Bioflavonoid*; Micronutrient; Nutrient*; Food Supplement*; Meal Replacement*; Nutrition Supplement*; Health Supplement*; multivitamin; omega 3; fish oil*; alpha lipoic acid; eicosapentaenoic; docosahexaenoic; fatty acid* amino acid*; taurine; cysteine; probiotic* |
| *Comparator* | Randomised; Randomized; Controlled; Control Group; Intervention; Adjunc*; Adjuvant; Augmentation; Supplement*; open label; single arm; feasibility; pilot; prospective; trial; |
| *Outcomes* | (All) |

Supplement 2. Cochrane risk of bias summaries for included studies.
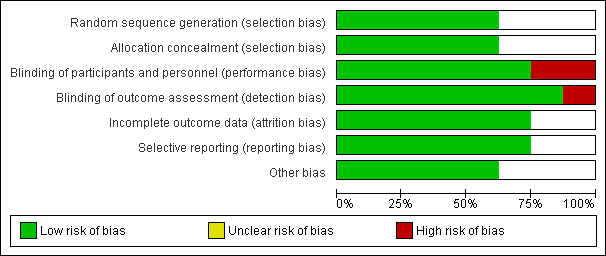


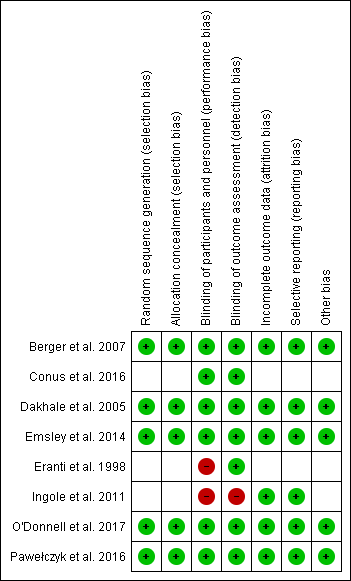

Supplement: Supplementary file 1 — Appendix S1. Systematic search algorithm. Appendix S2. Cochrane risk of bias summaries for included studies. [file EIP-12-774-s001.docx]
